# Empirical Model of Radio Wave Propagation in the Presence of Vegetation inside Greenhouses Using Regularized Regressions

**DOI:** 10.3390/s20226621

**Published:** 2020-11-19

**Authors:** Dora Cama-Pinto, Miguel Damas, Juan Antonio Holgado-Terriza, Francisco Manuel Arrabal-Campos, Francisco Gómez-Mula, Juan Antonio Martínez-Lao, Alejandro Cama-Pinto

**Affiliations:** 1 Department of Computer Architecture and Technology, University of Granada, 18071 Granada, Spain; mdamas@ugr.es (M.D.); frgomez@ugr.es (F.G.-M.); 2 Software Engineering Department, University of Granada, 18071 Granada, Spain; jholgado@ugr.es; 3 Department Engineering, University of Almeria, Ctra. Sacramento, s/n, 04120 La Cañada, Spain; fmarrabal@ual.es (F.M.A.-C.); jml357@ual.es (J.A.M.-L.); 4 Faculty of Engineering, Universidad de la Costa, Calle 58 # 55–66, 080002 Barranquilla, Atlántico, Colombia

**Keywords:** wireless propagation model, precision agriculture, COST235, FITU-R, ITU-R, Weisbberger model, propagation model, regularized regressions

## Abstract

Spain is Europe’s leading exporter of tomatoes harvested in greenhouses. The production of tomatoes should be kept and increased, supported by precision agriculture to meet food and commercial demand. The wireless sensor network (WSN) has demonstrated to be a tool to provide farmers with useful information on the state of their plantations due to its practical deployment. However, in order to measure its deployment within a crop, it is necessary to know the communication coverage of the nodes that make up the network. The multipath propagation of radio waves between the transceivers of the WSN nodes inside a greenhouse is degraded and attenuated by the intricate complex of stems, branches, leaf twigs, and fruits, all randomly oriented, that block the line of sight, consequently generating a signal power loss as the distance increases. Although the COST235 (European Cooperation in Science and Technology - COST), ITU-R (International Telecommunications Union—Radiocommunication Sector), FITU-R (Fitted ITU-R), and Weisbberger models provide an explanation of the radio wave propagation in the presence of vegetation in the 2.4 GHz ICM band, some significant discrepancies were found when they are applied to field tests with tomato greenhouses. In this paper, a novel method is proposed for determining an empirical model of radio wave attenuation for vegetation in the 2.4 GHz band, which includes the vegetation height as a parameter in addition to the distance between transceivers of WNS nodes. The empirical attenuation model was obtained applying regularized regressions with a multiparametric equation using experimental signal RSSI measurements achieved by our own RSSI measurement system for our field tests in four plantations. The evaluation parameters gave 0.948 for R^2^, 0.946 for R^2^ Adj considering fifth grade polynomial (20 parameters), and 0.942 for R^2^, and 0.940 for R^2^ Adj when a reduction of parameters was applied using the cross validation (15 parameters). These results verify the rationality and reliability of the empirical model. Finally, the model was validated considering experimental data from other plantations, reaching similar results to our proposed model.

## 1. Introduction

The wireless sensor networks (WSN) are an integral part of the Internet of Things (IoT) that connects the digital world with the real world [1,2]. It is usually used for low-cost applications in outdoor environments [3,4] to study the variability in the collection of environmental parameters for precision agriculture [5,6], such as temperature, humidity, soil nutrients, etc., deployed in vegetation environments [7,8]. The nodes are equipped with a microprocessor, transceiver, energy unit, and sensors that collect, process, and store data [9].

The adoption of this technology in precision agriculture [10] will help to improve agricultural yields by counteracting the negative effects of climate change, the reduction in the area of land used for agriculture, and the reduction of fresh water, among others [11,12]. Increasing agricultural production will provide essential elements for humans [13] because the world’s population will grow by 31% by 2050 [14]. According to the Food and Agriculture Organization of the United Nations, feeding the Earth’s growing population will require producing 70% more food in 2050 than in 2006 [15].

Precision agriculture (PA) is defined as the management of spatial and temporal variability in agriculture through the use of information and control technologies for the collection of data with higher resolution than those obtained from remote sensing, laboratory tests, etc. [16]. The WSN, part of the PA, provides real-time information on crop land that helps farmers to make decisions and implement practices in a timely and correct manner in the field [13,17]. For example, mitigating water scarcity, saving water, and being more efficient in agricultural irrigation, which accounts for approximately 70% of the world’s total fresh water [18,19], by reliably measuring soil moisture in real time over large agricultural areas [20]. In fact, the plains of the Almeria province represent one of the greatest potential for the use of PA in Spain. It is located in the southeast of the Iberian Peninsula and has the largest concentration of greenhouses in the Mediterranean basin (>31,000 ha) [21] and in the world [22]. This region has the largest area of landless vegetable production in the country and is the leading producer of tomatoes in Spain [23,24], followed in descending order by sweet peppers and cucumbers [25].

Given this perspective and the potential usefulness of WSN in the PA, the propagation path loss of radio waves was analysed to ensure the reliability of wireless communication in the coverage of the sensor nodes distributed in a tomato greenhouse in Almería, Andalusia, Spain. The values obtained through field tests are compared with empirical models, such as Weissberger and ITUR [26], but many discrepancies can be found because, in general, these models only take account the distance between transceivers of WSN nodes. In order to improve the current accepted empirical models specifically for tomatoes greenhouse we elaborate our own empirical model by means of regularized regressions taking into account the height in addition to the distance.

The most important contributions of this article are the following:

- A new empirical model of radio wave attenuation for vegetation is developed for the 2.4 GHz band which includes the vegetation height as a parameter to be taken into account in addition to the distance between the transmitter and receiver node.
- This attenuation model has been obtained from attenuation measurements made with our own RSSI measurement system from four tomato greenhouses. The proposed model has then been validated with experimental data from other plantations.
- A method is presented to determine the signal attenuation in an empirical way that can be applied to other greenhouse plantations.
- The research contributes to more accurate measurements of attenuation due to vegetation that allows a better planning of the deployment of WSN nodes within a tomato greenhouse for, e.g., humidity control.

Section 2 discusses the state of the art and works related to our research. Section 3 details the empirical models that are used for the calculation of radio propagation in vegetation presence. Section 4 explains the experimental procedure used to record values of radio wave attenuation when passing through vegetation. Section 5 proposes a method to determine radio wave attenuation in the presence of vegetation. Section 6 shows the results obtained and analyses them after comparing our model with real measurements in tomato greenhouses. The next section explains the advantages of our proposed model compared to the classical empirical models of radio propagation in vegetation environments. Finally, in the last section, we present the conclusions and possible future works.

## 2. State of the Art and Related Works

The multipath propagation of radio waves between the transceivers of the WSN nodes [7,27] is degraded due to propagation loss [13]. The incident electromagnetic field is attenuated by diffraction, scattering, reflection of the intricate complex of stems, branches, leaf twigs, and fruits, all randomly oriented [28,29], that block the line of sight (LOS) [13] in a greenhouse, consequently generating a signal power loss. The calculation of this loss determines the reliability of the wireless link along with the success of the target application of the WSN deployment [6,30]. Their knowledge allows for the creation of more appropriate models in the design of wireless communication networks [31]. In our study, we modeled radio wave propagation from the received signal strength indicator (RSSI), a measurement obtained through the received power of the wireless signals at different transmission distances and different antenna heights inside a tomato greenhouse [11]. This serves to establish the maximum effective distance between nodes and to predict the number of sensors needed to cover the deployment of WSN in a crop area [7]. 

Analyzing relating researches we can find similar works that characterize the influence of vegetation when it is traversed by a radio wave with a frequency of 2.4 GHz by a mango greenhouse [32], green pepper greenhouse [33], and a plum orchard [34]. These studies showed that the attenuation of the radio wave varies with the height and type of vegetation it passes through. Empirical models of radio wave propagation in the presence of vegetation vary considerably from the measurements taken in field tests, as is the case in [35] where a new model based on linear regression had to be developed in a mango greenhouse, or the study in [26] where cubic regression was used to define a model of attenuation in tomato greenhouses. 

The modelling of electromagnetic wave propagation is an essential tool for wireless network design and radio frequency (RF) interference studies [36]. It can be divided into two categories: empirical (or statistical) and deterministic (analytical) models. Empirical models are more widely used to solve practical problems, relying on actual measurements of radio frequency on communication channels. Such models are simple to apply and can offer a quick solution. However, their predictions are not always accurate. Unlike empirical models, deterministic models of greater complexity, are based on numerical approaches to Maxwell’s equations that significantly improve the reliability of predictions [37,38]. Statistical models were originally developed to provide estimates without field data [39]. Empirical models based on regression methods are commonly used for cover estimates, depending on the type of vegetation where the measurements were made [35,40]. The most commonly used radio propagation models for the design of wireless networks in the presence of vegetation are the empirical ones (see Table 1). Therefore, a method of moderate complexity that provides greater accuracy and generality than empirical models is still needed to estimate the electromagnetic attenuation that passes through vegetation [41].

The empirical models of attenuation by vegetation defined in Table 1 depends on the radio wave frequency and the distance in the depth of the vegetation. However, all of them give an estimate of the attenuation of the signal that is quite different from the one obtained in the field tests. This is the reason why, in this paper, we present a novel general method to determine an empirical model of the attenuation by vegetation.

## 3. Experimental Determination of Attenuation in Vegetation

An accurate determination of the signal attenuation in vegetation is significant in precise agriculture to know how the WSN nodes should be deployed in a greenhouse. The attenuation can be calculated by the usage of a RSSI measurement system that provides an accurate measurement of the signal strength between a transmission node and a receiver node at different distances and heights. In this case, our own development of the RSSI measurement system was applied in this study. 

### 3.1. RSSI Measurement System

**Figure 1:**
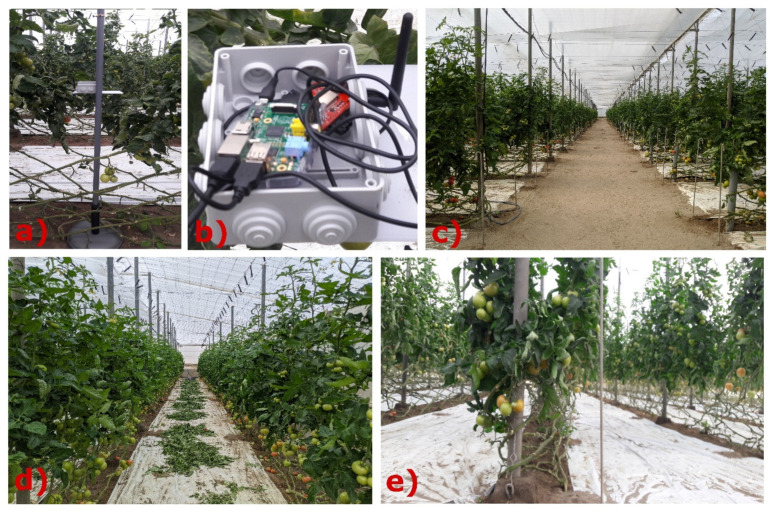
Images of the greenhouse. (**a**) Remote node that sends the signal to the sink node (TX). (**b**) Equipment used by RSSI measurement system in the receiver - RX (Re-Mote connected with USB cable to the Raspberry Pi 3) **(c)** Main aisle (**d**) Side aisle between two “tomato plant walls” (**e**) Thickness view of the “tomato plant walls”.

**Figure 2:**
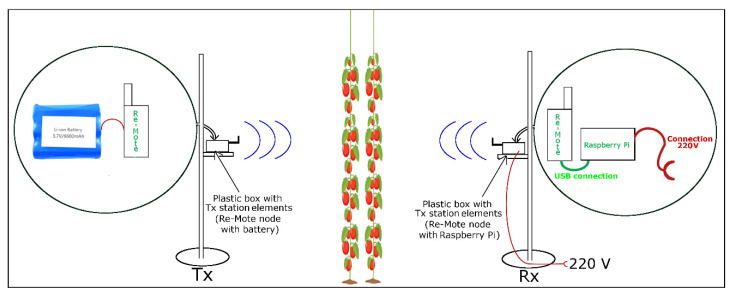
Deployment of the two TX and RX stations communicating wirelessly in a cross section of a tomato greenhouse.

Our system is divided into two parts. The first one is the node that sends the signal (TX node) (Figure 1a) and the second one is the receiver (RX) (Figure 1b) which consists of a wireless node (RX node) that collect the signal sent by the TX node and an embedded computer that stores the power signal received in dBm. TX and RX devices are supported by a mast on a 17-kg base (See Figure 1a), to avoid trembling movements that affect the measurement and radio link stability. The following hardware and software elements are used to carry out field measurements of radio wave attenuation in the 2.4 GHz frequency band in the presence of vegetation inside a greenhouse:

(1) The deployment nodes, both on the transmitting (TX) and receiving side (RX), are the Re-Mote with the technical characteristics described in [43]. They work with the Contiki Operating System and their applications are written in C language. The nodes communicate in the 2.4 GHz industrial scientific medical (ISM) band (λ = 12.24 cm), with the IEEE802.15.4 standard that defines the media access control (MAC) and physics (PHY) layer, designed for low energy consumption, low speed and short-range technologies [44,45]. It has a 3-dBi gain dipole antenna, the receiver sensitivity is −100 dBm with omnidirectional radiation pattern, i.e., the radiated signal has the same intensity in all directions [13]. Figure 2 describes the TX node powered by a 3.7 V Li-ion battery with capacity of 6600 mAh, which gave it autonomy of operation because only it moved away from the RX node. These elements are enclosed in a PVC box with an IP65 protection rating, prepared to prevent dust and water jets from entering. In Figure 2 we can see that the electrical energy with which the RX node operated was obtained from its connection to the Raspberry Pi USB port. In addition, in Figure 2, we see that this embedded computer was powered through a 220 V electrical outlet in the greenhouse. There are other studies on this subject, which also use wireless sensor networks (WSN), and use modular devices based on Arduino boards and Xbee radio modules [10]. However, the authors prefer to use the Re-Mote nodes due to it being a small device (7.3 × 4 cm), with integrated radio modules in the 2.4 GHz, 868/915 MHz frequency bands, in addition to having previous knowledge of their configuration.
(2) The RSSI information collected in the sink node is forwarded to the Raspberry-Pi 3 embedded computer and stored in its microSD memory in CSV format to facilitate its processing. The Raspberry Pi performs its functions through the Raspbian operating system. It physically communicates with the Re-Mote sink node through the USB port and receives the RSSI values by executing the script written with the Python programming language.

### 3.2. Deployment and Field Testing

**Figure 3:**
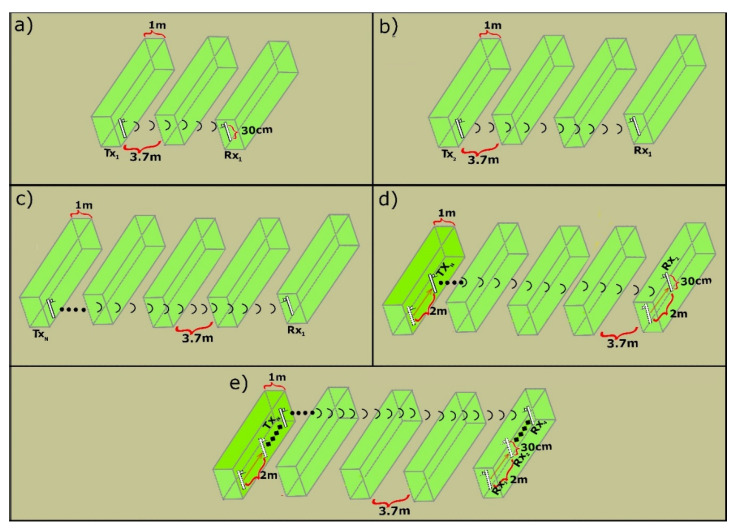
Diagram of measurements stages inside the greenhouse. (**a**) Wireless link arrangement between TX and RX nodes when there is one (1) "tomato plant wall" between them (**b**) Wireless link arrangement between TX and RX nodes when there are two (2) "tomato plant walls" between them (**c**) Arrangement of wireless link between TX and RX nodes when there are three "tomato plant walls" between them (**d**) Change of position at two (02) meters from both TX and RX nodes along the aisle (**e**) Change up to four (4) positions along each aisle of the TX (TX_1_, TX_2_, TX_3_, TX_4_) and RX (RX_1_, RX_2_, RX_3_, RX_4_) nodes.

Our field tests were carried out in four tomato greenhouses with dimensions of 1000 m^2^, on flat ground, in four locations in the province of Almeria (Spain): (a) La Cañada–8 m.a.s.l, (b) Retamar–12 m.a.s.l., (c) El Alquian–42 m.a.s.l., (d) Níjar–248 m.a.s.l. All of them are close to the Alboran Sea, with the exception of the one located in Níjar, as it is the closest to the mountain. The greenhouses have a main aisle (Figure 1c) and the side aisles (Figure 1d) that are separated from each other by the "tomato plant walls (Figure 1e)". The steps for the tests were as follows in each greenhouse:

(a) The nodes are placed at the same height. The sink node receives the value of the attenuated signal. The sink node receives the value of the attenuated signal sent by the remote node every 5 min, repeating this measurement 10 times. It is located at the end of the greenhouse and another node behind the first wall of tomato plants, which transmits the signal (TX node) as shown in Figure 3a. In other words, a wall of tomato plants between the nodes blocks the signal.
(b) The sink node remains in its same position RX_1_, while the other node, the transmitter (TX), moves away in a straight line to two tomato plant walls in the TX_2_ position described in Figure 3b, thus successively increasing the number of “tomato plant walls” and reach the final position: TX_N_, increasing the distance between both nodes (TX and RX), as well as the attenuation of the signal detailed in Figure 3c.
(c) When it reaches the limit of coverage between the two nodes, the process is restarted, but moving the nodes 2 m along the “tomato wall”, with the receiver node now in position RX_2_ as shown in Figure 3d, and then repeat the two previous steps. Thus, in Figure 3e, the sink node called “RX_1_” moves like this to “RX_4_” passing previously through the positions “RX_2_” and “RX_3_”, each one receiving the attenuated signal sent by the transmitter nodes from TX_1_ to TX_N_. 160 RSSI values are recorded for each specific distance between the TX and RX nodes, 10 values in each position of the sink node RX_1_, RX_2_, RX_3_, RX_4_ and this process is carried out in 4 different greenhouses. The collected values are averaged.
(d) The steps shown above are repeated for new heights. The communication between the nodes was evaluated when the height of their antennas was 30 cm, 50 cm, 70 cm, 90 cm, 100 cm, 150 cm, and 200 cm.

### 3.3. Attenuation of Radio Waves in Free Space

**Figure 4:**
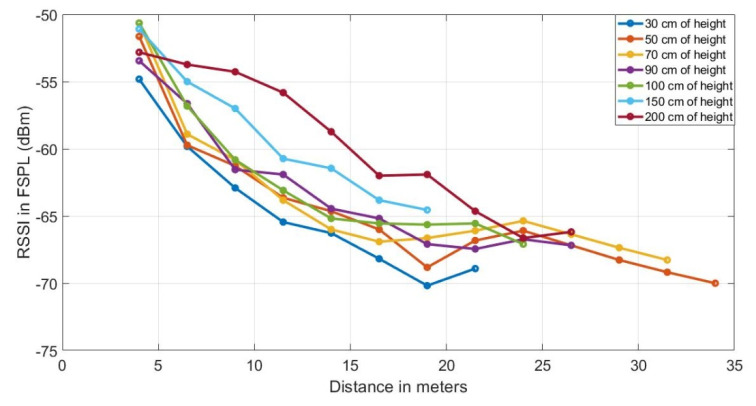
Received signal level average in dBm of the four locations, without line of sight obstructed (LOS) between transmitter (TX) and receiver (RX) nodes at different heights from the ground (30 cm, 50 cm, 70 cm, 90 cm, 100 cm, 150 cm).

The attenuation of radio waves in free space is calculated from the equation $L_{fspl}=10log_{10}{\left(\frac{\lambda }{4\pi d}\right)}^{2}$ [38], being on a logarithmic scale: $L_{fspl}\left(dB\right)=20log_{10}\left(f\right)+20\log\left(d\right)-147.56$ (*f* in Hz, *d* in meters) with line of sight (LoS). Figure 4 is the received power measurement obtained without line-of-sight obstruction (P_r_fspl_), the attenuation of the received signal is caused only by $L_{fspl}$.

### 3.4. Received Signal Strength in the Presence of Vegetation

**Figure 5:**
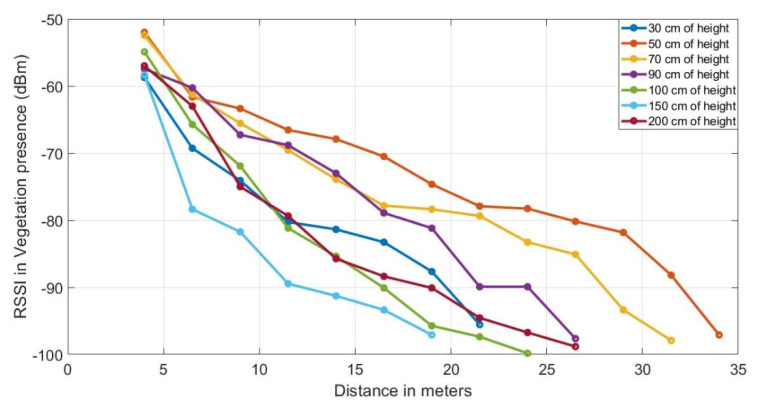
Received signal level average in dBm of the four locations, with line of sight obstructed by tomato plant walls between transmitter (TX) and receiver (RX) nodes at different heights from the ground (30 cm, 50 cm, 70 cm, 90 cm, 100 cm, 150 cm).

The measurements of the powers received in the sink node (RX), attenuated by the presence of vegetation is $P_{rx\_foliage}$. These values are recorded according to the deployment steps shown in Figure 3, and are shown in the curve graph in Figure 5. Here, we observe that greater coverage is achieved between the TX and RX nodes when the heights of the antennae of the nodes are 50 cm from the ground, with a maximum distance between them of 35 m.

### 3.5. Calculation of Attenuation by the Presence of Vegetation

**Figure 6:**
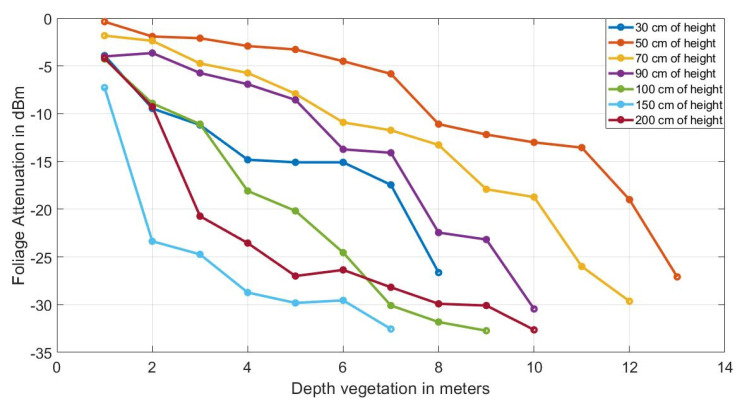
Attenuation average in (dB) of the four locations by foliage presence between transmitter (TX) and receiver (RX) nodes at different heights from the ground (30 cm, 50 cm, 70 cm, 90 cm, 100 cm, 150 cm).

Equation (3) provides the attenuation caused by the foliage (Lfoliage), it is obtained by subtracting $P_{rx\_foliage}$ (Equation (2)) with the power reception when the signal travel in free space path loss $P_{rx\_fspl}$ (Equation (1)) [28], the result can be seen in the graph of curves in Figure 6, where the X axis indicates the depth of the thickness of the vegetation in sections that increase by one meter as this is the width of the wall of each “tomato tree wall”. Below, $P_{tx}$ denotes transmit power, $G_{tx}$ transmit antenna gain, and $G_{rx}$ receive antenna gain.

(1) $$P_{rx\_fspl}\left(dBm\right)=P_{tx}+G_{tx}+G_{rx}-L_{fspl}$$

(2) $$P_{r\_foliage}\left(dBm\right)=P_{tx}+G_{tx}+G_{rx}-L_{fspl}-L_{foliage}$$

(3) $$L_{foliage}\left(dB\right)=P_{rx\_foliage}\left(dBm\right)-P_{rx\_fspl}\left(dBm\right)$$

**Figure 7:**
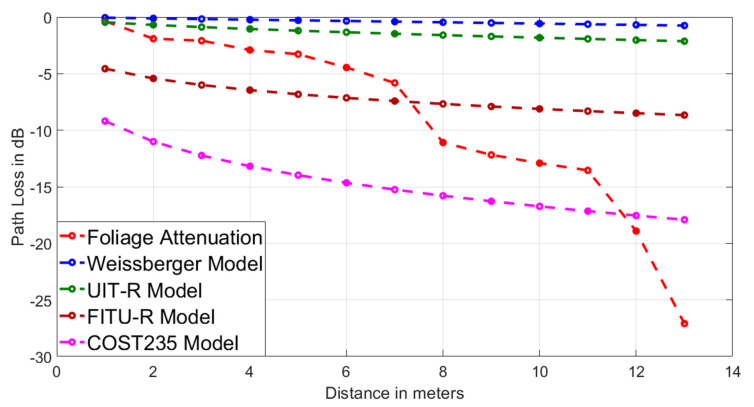
Foliage attenuation average in (dB) of the four locations at 0.5 m from the ground vs. Empirical Models (Weissberger, UIT, FITU-R, COST235).

The foliage attenuation curve ($L_{foliage}$) can be compared with empirical models of: Weissberger, ITU-R, FITU-R, COST235. As shown in Figure 7, none of them resembles the value obtained empirically, so the need to find a new model arises.

## 4. Proposed Method for Determining the Attenuation by the Presence of Vegetation

### 4.1. Parametric Optimization of the Attenuation

For this process, we use the average L_foliage in the measurements at different distances between the TX and RX with the heights of the antennas 30 cm, 50 cm, 70 cm, 90 cm, 100 cm, 150 cm, and 200 cm in each location (La Cañada, Retamar, El Alquian, Níjar). Finally, the development of the proposed model is obtained using the average of the L_foliage value in all the locations for a specific height and distance (69 values in total). The empirical models considered until now could only determine the attenuation of the signal as a function of frequency and distance. In this article, we determine this attenuation also taking into account the height of the node antennas. For this purpose, we look for a polynomial equation based on the exponential decay model (EDM), the Weissberger model, and the receiver height described in Equation (4),

(4) $$L_{foliage}\left(d,h\right)=\theta_{1}+\theta_{2}f^{\theta_{0}}d^{\theta_{3}}h^{\theta_{4}}+\overset{20}{\underset{k=1}{\sum }}\theta_{k}d^{i}h^{j}\;\forall \;i,j=\left\{0,1,2,\ldots ,5\right\}$$

where the parameters are $\left\{\theta_{1},\theta_{2},\ldots ,\theta_{k}\right\}$, $f^{\theta_{0}}$ is the value of the emission frequency, this value is constant because we evaluate in the 2.4 GHz central frequency ISM band, d is the value of the distance between transmitter and receiver, and h is the value of the heights of the receiver and transmitter. Equation (5) was developed for 20 parameters leaving it this way,

(5) $$\begin{array}{cc} L_{\text{foliage}}(d,h)= & \theta_{1}+\theta_{2}d^{\theta_{3}}h^{\theta_{4}}+\theta_{5}d^{2}h+\theta_{6}dh^{2}+\theta_{7}d^{2}h^{2}+\theta_{8}d^{3}h+\theta_{9}d^{3}h^{2}\\ & +\theta_{10}d^{3}h^{3}+\theta_{11}d^{2}h^{3}+\theta_{12}dh^{3}+\theta_{13}d^{2}+\theta_{14}h^{2}+\theta_{15}d^{3}+\theta_{16}h^{3}\\ & +\theta_{17}d^{4}+\theta_{18}h^{4}+\theta_{19}d^{5}+\theta_{20}h^{5}x \end{array}$$

$L_{foliage}$ has been developed for fifth grade polynomial. The selection of the polynomial grade addresses the need to have a sufficiently large grade to explain the physical behaviour of the attenuation. A polynomial of grade 5 (i,j from 0 to 5) is sufficiently large to explain this physical behaviour of attenuation, verifying this statement by the small error achieved, then proceed to eliminate terms by simplifying the equation. The parameters of Equation (8) are fitted using Equation (6), function cost  J(θ). To accomplish this optimization, it is used the optimization algorithm based on Nelder–Mead simplex method [46]

The multiparametric equation has been obtained by optimizing the following regularization problem,

(6) $$\underset{\theta \in R^{n}}{\min}{\left\|L_{foliage}\left(\theta \right)-dB\right\|}^{2}+\lambda {\left\|\theta \right\|}^{2}$$

where $L_{foliage}\left(\theta \right)$ is the attenuation as function of θ, *dB* is the measured values, λ>0 is the regularization parameter, $\|\cdot \|={\left\|\cdot \right\|}_{2}$ is the Euclidian norm. The number of experiments taken that corresponds to 69 averaged values (from the four locations). $L_{foliage}\left(\theta \right)$ is the model represented in Equation (4).

The prevention of the over-adjustment of variables is carried out by means of a regularising term called Tikhonov’s regularisation [47,48,49], where λ is the regularising term and determines the weight that the parameters should have in the cost function. This type of problem ensures that there is no over-adjustment in the parameterised functions. To this optimization problem, it is necessary to calculate the gradients for each direction. Equation (7) shows how these gradients can be calculated.

(7) $$J\left(\theta \right)={\left\|L_{foliage}\left(\theta \right)-dB\right\|}^{2}+\lambda {\left\|\theta \right\|}^{2}\;\frac{\partial J\left(\theta \right)}{\partial \;\theta_{1}}=2\|L_{foliage}\left(\theta \right)-dB\|\frac{\partial L_{foliage}^{\left(m\right)}}{\partial_{1}}+2\lambda \|\theta \|\;\frac{\partial J\left(\theta \right)}{\partial \;\theta_{2}}=2\|L_{foliage}\left(\theta \right)-dB\|\frac{\partial L_{foliage}^{\left(m\right)}}{\partial_{2}}+2\lambda \|\theta \|\;\ldots \;\frac{\partial J\left(\theta \right)}{\partial \;\theta_{20}}=2\|L_{foliage}\left(\theta \right)-dB\|\frac{\partial L_{foliage}^{\left(m\right)}}{\partial_{20}}+2\lambda \|\theta \|$$

**Figure 8:**
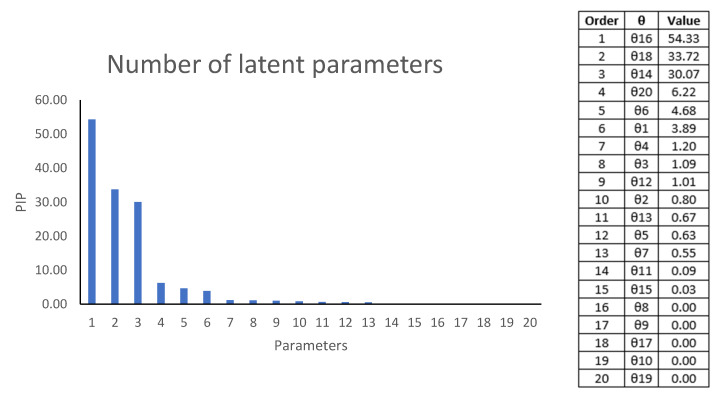
PIP values for the chosen model. Values lower than 0.03 are excluded from the model, so the parameters $\theta_{8},\theta_{9},\;\theta_{17},\;\theta_{10}\;and\;\theta_{19}$ are excluded from the model.

The values taken by the parameters that determine their importance, called “projected parameter importance” (PIP). Parameter values less than 0.03 are considered not to contribute to the model. The determined PIP is represented in the following Figure 8. Taking into account the previous criterion, it can be observed that some parameters hardly contribute to the $L_{foliage}$ function. These parameters are $\theta_{8},\theta_{9},\;\theta_{17},\;\theta_{10}\;y\;\theta_{19}$.

**Figure 9:**
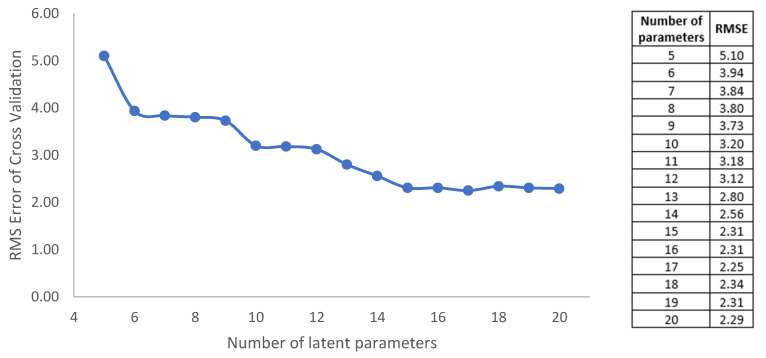
Root mean squared error of cross validation (RMSECV) as a function of number of parameters used in the $L_{foliage}$ model.

The multiparametric equation has been reduced using the PIP parameters for those greater than 0.03. The quadratic validation mean square error (RMSECV) has been applied for the reduction of latent parameters in order to have a reduced equation without losing information when the model is applied, as shown in Figure 9.

**Figure 10:**
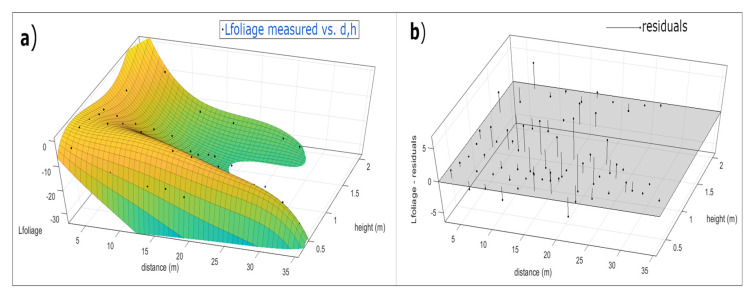
(**a**) Measured values represented by black dots together with the optimized $L_{foliage}\left(d,h\right)$ model for 20 parameters. (**b**) Residual values of $L_{foliage}\left(d,h\right)$ determined as difference between measured data and predicted data.

Figure 10 shows the solution obtained for the 20 parameters set. This figure shows a 3D view of the Equation (4) (Figure 10a), where the x-axe is the variable distance (d) and the *y*-axis is the variable height (h), both in meters. The z-axe are the values when the function $L_{foliage}\left(d,h\right)$ is evaluated for each value of distance and height. The black dots are the measured values in the greenhouse. Figure 10b shows the residual values between the measured data and the predicted data. In Table 2, we can see the values of the optimized parameters.

The quality of this model has been expressed by the cross validation of parameters, R^2^ and Q^2^, which represent the explained variability, giving values of 0.948 and 0.938 respectively. Also, the RMSECV (mean square error of quadratic validation) has been used as an index, whose value is 2.29. The model has been validated by the permutation test. 

It is used as a model evaluation, the mean square error (MSE), the root of the mean square error (RMSE) and the mean absolute percentage error (MAPE). In addition, the Akaike information criterion (AIC) is provided, indicating the loss of information in the model considered and the Schwarz information criterion (SBC) which establishes the goodness of fit with the estimated parameters. Both the AIC and SBC are comparative indices that must be compared with other models. They will be used later to check that there is no loss of information in the estimate. The evaluation values of the optimized multiparametric function are shown in Table 3, where the adjusted R^2^ value is 0.946.

### 4.2. Reduced Parametric Optimization of the Attenuation

The reduction of Equation (5) can be deduced using Figure 8 and Figure 9. Observing Figure 9 in detail, from 15 parameters used the RMSECV does not vary. Therefore, in Figure 8, we observe that the parameters close to zero that must be cancelled are: $\theta_{8},\theta_{9},\;\theta_{17},\;\theta_{10}\;y\;\theta_{19}$, because they do not contribute any quantity to the $L_{foliage}$ model. Equation (5) can be reduced in the following Equation (8),

(8) $$L_{foliage}\left(d,h\right)=\theta_{1}+\theta_{2}d^{\theta_{3}}h^{\theta_{4}}+\theta_{5}d^{2}h+\theta_{6}dh^{2}+\theta_{7}d^{2}h^{2}+\theta_{11}d^{2}h^{3}+\theta_{12}dh^{3}\;\;+\theta_{13}d^{2}+\theta_{14}h^{2}+\theta_{15}d^{3}+\theta_{16}h^{3}+\theta_{18}h^{4}+\theta_{20}h^{5}$$

where:

(9) $$L_{foliage}\left(d,h\right)=\theta_{2}d^{\theta_{3}}h^{\theta_{4}}+f_{compensation}\;$$

and:

(10) $$\begin{array}{cc} f_{compensation}= & \theta_{1}+\theta_{5}d^{2}h+\theta_{6}dh^{2}+\theta_{7}d^{2}h^{2}+\theta_{11}d^{2}h^{3}+\theta_{12}dh^{3}+\theta_{13}d^{2}+\theta_{14}h^{2}\\ & +\theta_{15}d^{3}+\theta_{16}h^{3}+\theta_{18}h^{4}+\theta_{20}h^{5} \end{array}$$

Equation (9) has a first term equivalent to the attenuation model given by Weissberger and a second term $f_{compensation}$ that determines the compensation to the Weissberger model that has to be added in order to estimate $L_{foliage}$. Taking into account Figure 8 and Figure 9, the parameters $\theta_{5},\theta_{7},\theta_{11},\theta_{13},\theta_{15}$ of Equation (8) could be ignored in the model because of their slight root mean square error (RMSE). As expected, this error would be around 3.12, meaning that the model does not depend on distance when varying the height. However, we only override those parameters that have an approximate value of zero, these parameters are $\theta_{8},\theta_{9},\;\theta_{17},\;\theta_{10}\;y\;\theta_{19}$. The application of the parametric function is applied to adjust experimental values obtained by Equation (3). The parameters of the Equation (8) are fitting using the function cost  J(θ)*,* Equation (6). To acomplish this optimization, it is used the optimization algorithm based on Nelder–Mead simplex method [46]

**Figure 11:**
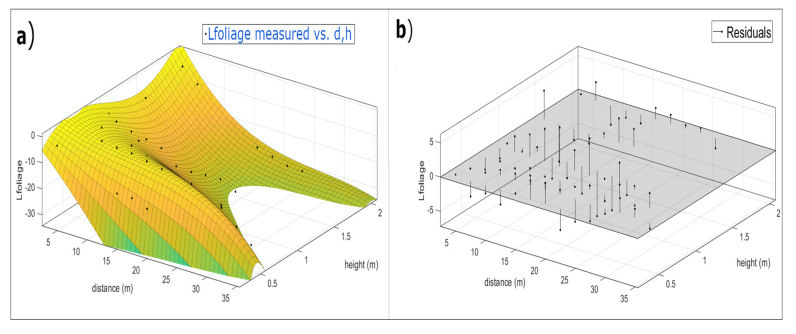
(**a**) Measured values represented by black dots together with the optimized $L_{foliage}\left(d,h\right)$ model for 15 parameters. (**b**) Residual values of $L_{foliage}\left(d,h\right)$ determined as difference between measured data and predicted data.

Figure 11 shows the solution obtained for the 15 parameters set. This figure shows a 3D view of the Equation (4) (Figure 11a), where the x-axe is the variable distance (d) and the *y*-axis is the variable height (h), both in meters. The z-axe are the values when the function $L_{foliage}\left(d,h\right)$ is evaluated for each value of distance and height. The black dots are the measured values in the greenhouse. Figure 11b shows the residual values between the measured data and the predicted data. In Table 4, we can see the values of the optimized parameters.

The quality of this new model is expressed by the cross validation of parameters, R^2^ and Q^2^, in order to make the comparison with the previous model. The RMSECV is worth 2.40. The model has also been validated by the permutation test.

As with the previous model, the mean square error (MSE), the root of the mean square error (RMSE) and the mean absolute percentage error (MAPE) have been used as assessments. In addition, the Akaike information criterion (AIC) and the Schwarz information criterion (SBC) are provided, indicating a good fit with the estimated parameters. The evaluation values of the optimized multiparametric function are shown in Table 5. The adjusted R^2^ value is 0.940.

The results of the radio wave attenuation models for vegetation in a tomato greenhouse developed in this research are very similar, the R^2^ for both are only 0.004, establishing that the correlation between the model and the data obtained is 0.942 for the first and 0.946 for the second model. Also, the adjusted R^2^, in both cases, is very similar, differing by only 0.006. The values of MSE and RMSE show hardly any significant differences. The ASM, the ACI, and the SBC belong to the same order of magnitude, so it follows that there is no loss of information when one model or the other is applied.

Both 15 and 20 parameter models are valid. In both cases, good evaluation indicators are obtained so that either of them can be used. However, the 15-parameter model has an advantage over the 20-parameter model. This advantage is that it uses five fewer parameters than the 20-parameter model (see Table 6).

## 5. Results and Analysis

The model created is contrasted with the experimental measurements taken from the four greenhouses in February 2020 and the measurement taken in February 2018 at the La Cañada greenhouse. The values summarised in Table 7 reveal that the model obtained is well suited to tomato greenhouses, regardless of the sea level where it is located.

In order to evaluate the generalized error, the model was evaluated with real values for fitting the model. Therefore, in the Table 7 shows the evaluation parameters for other greenhouses measurements. On the one hand, the R^2^ parameter is between 0.921 and 0.895. The generalized R^2^ can be considered as their average. The R^2^ average is 0.906. On the other hand, the RMSE parameter is between 3.389 and 2.957. The generalized RMSE can be considered as their average. The RMSE average is 3.156. This error is greater than the error obtained using the values in the fitting model, something that it is expected. 

**Figure 12:**
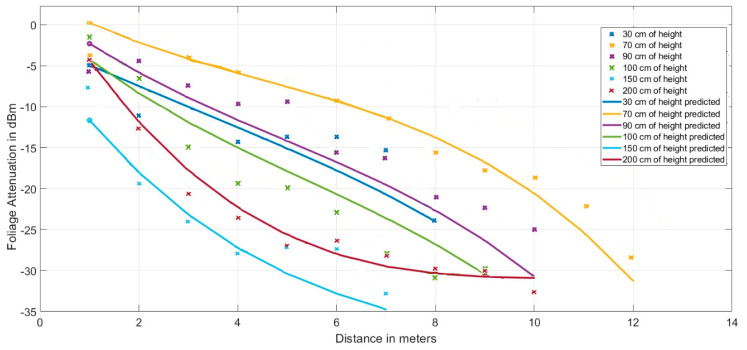
Curves of the proposed model vs. values taken in field tests in the year 2018.

The values collected in the field tests in 2018 in the tomato greenhouse of La Cañada, in Almería (Spain), represented with dots, were contrasted to the equation of the proposed model (coloured curves) in the graph of Figure 12. As expected, the proposed model fits adequately with the experimental data. 

The results prove the validity of the new method, and the possibility of using it for planning the deployment of WSN nodes in different types of tomato greenhouses. In addition, with the field tests, it is determined that the most favorable height to achieve the greatest coverage between TX and RX nodes is 0.5 m from the surface. With the proposed equation, we established the behavior model of the radio wave attenuation in its passage through the foliage in the 2.4 GHz band widely used by different standards or technologies, such as ZigBee, WiFi, IEEE 802.15.4, and IEEE 802.15.1 among others.

## 6. Discussion 

Multi-hop mesh structures between the nodes of wireless sensor networks are particularly useful in agricultural surveillance systems because of their simplicity of application. The evaluation of path losses caused by foliage is relevant to network design and planning. Vegetation propagation models, such as ITU-R, FITU-R, and COST 235, are currently available, but these differ considerably from empirical models when compared with field test measurements [50]. This has been the reason why attempts are made to improve such models through linear regressions as in research done in mango greenhouses [47]. Radio wave attenuation differs from vegetation height because the distribution of foliage in a plant varies at different distances from the ground as evidenced in orchard studies [48]. Therefore, the importance of our work is because we deliver a model that uses the height variable giving better precision in the behaviour of the radio wave when it passes through a tomato greenhouse in the 2.4 GHz band at different distances as well as the mathematical procedure to arrive at this model equation.

## 7. Conclusions and Future Work

Radio wave attenuation measurements were taken experimentally inside a tomato greenhouse using a network of wireless sensors sending a signal in the 2.4 GHz ISM band. The recorded attenuation data are compared with the values of existing empirical vegetation attenuation models, giving considerable margin of error between them. 

In that sense, to improve the predictability of the current empirical models we developed our own empirical model using the reduction of variables by means of a regularized regression method, confirming its usefulness because we ensure that there is no overfitting in the estimation of the parameters. A reduction of parameters is carried out to ensure that the new model found is not over parameterized. 

Our empirical model, based on the modified exponential decay model (EDM), has the simplicity of only taking the distance between the nodes as a variable and also includes the height of their antennas (transmitting and receiving) unlike Weissberger’s model because, as expected, the attenuation is impacted more by the height variable than by the distance variable. 

The evaluation parameters of the empirical model yield robustness and reliability. The first approach obtained high score for R^2^ and R^2^ Adj, 0.948 and 0.946, respectively. As well, the reduced model got 0.942 for R^2^ and 0.940 for R^2^ Adj. Finally, the generalized error yielded 0.906 for R^2^ and 0.902 for R^2^ Adj. All these results are expected. 

The methodology presented will serve to develop new models of radio wave attenuation in the presence of other types of crops, where the attenuation function of the foliage is due to two terms, the first being an exponential attenuation and the second depending on a compensation function related to the environment where the signal is propagated. In future studies, it is important to investigate the compensatory part of our proposed modified equation obtained from the modified exponential decay model (MED) to implement new empirical models, but also to analyse how the model will behave if the device moves according to plant growth.

The results obtained with the developed model are beneficial in precision agriculture, because it allows to determine the most suitable locations for the sensor nodes, and to determine their necessary number when planning their deployment so that the whole greenhouse can be monitored in terms of its conditions of temperature, environmental humidity, soil moisture, air quality, etc.

## Figures and Tables

**Table 1:** Empirical propagation models.

Model	Equation	Description	Frequency Range
The modified exponential decay model (EDM) is the generic empirical model [13,41]	L_MED_ = Af^B^d^c^ f = Frequency (MHz) d = Vegetation depth (m) B,C = Model parameters	*A, B, y C* are empirical constants. This model is suggested by the International Telecommunication Union (UIT)	30 MHz to 30 GHz
Weissberger [28,35,42]	L_Weiss_ = 0.45f^0.284^d^0^ m < d < 14 m L_Weiss_ = 1.33f^0.284^d^0.558^ 14 m < d < 400 m	f is the frequency in GHz and d is the depth of the vegetation in meters	230 MHz to 95 GHz
ITU-R [28]	L_ITU-R_ = 0.2*f*^0.3^ *d*^0.6^, d < 400 m.	f is the frequency in MHz and d is the depth of vegetation in meters	200 MHz to 95 GHz
COST235 [28,35,42]	L_COST235_ = 26.6*f^−0.2^d^0.5^out-of-leaf* L_COST235_ = 15.6*f^−0.009^d^0.26^in-leaf*	f is the frequency in MHz and d is the depth of vegetation in meters, d < 200 m	9.6 GHz to 57.6 GHz
Fitted ITU-R (FITU-R) Model [28,35,42]	L_FITU-R_ = 0.37*f^−0.18^d^0.59^out-of-leaf* L_FITU-R_ = 0.39*f^−0.39^d^0.25^in-leaf*	f is the frequency in MHz and d is the depth of vegetation in meters,	VHF to millimetric waves

**Table 2:** Parameter values optimized using the cost function of the Equation (6). Some parameters as it can be seen have values close to zero.

θ	1	2	3	4	5	6	7	8	9	10
**Value**	3.89	−0.80	1.09	−1.20	−0.63	−4.68	0.55	0.00	0.00	0.00
**θ**	**11**	**12**	**13**	**14**	**15**	**16**	**17**	**18**	**19**	**20**
**Value**	−0.09	1.01	0.67	30.07	−0.03	−54.33	0.00	33.72	0.00	−6.22

**Table 3:** Evaluation parameters of the regularised optimisation carried out for 20 parameters.

	Nº Parameters	R^2^	R^2^ Adj.	MSE	RMSE	MAPE	AIC	SBC
**$L_{foliage}\left(d,h\right)$**	20	0.948	0.946	5.27	2.29	0.167	410	199

**Table 4:** Parameter values that have been optimized using the cost function of the Equation (3).

θ	1	2	3	4	5	6	7	
**Value**	−6.73	−0.08	1.62	−1.59	−0.57	−3.33	0.49	
**θ**	**11**	**12**	**13**	**14**	**15**	**16**	**18**	**20**
**Value**	−0.12	1.10	0.30	100.59	0.00	−167.33	95.02	−17.69

**Table 5:** Evaluation parameters of the regularised optimisation carried out for 15 parameters.

	Nº Parameters	R^2^	R^2^ Adj.	MSE	RMSE	MAPE	AIC	SBC
**$L_{foliage}\left(d,h\right)$**	15	0.942	0.940	5.80	2.40	0.189	417	206

**Table 6:** Evaluation parameters comparison of the regularized optimization carried out for 15 and 20 parameters.

	Nº Parameters	R^2^	R^2^ Adj.	MSE	RMSE	MAPE	AIC	SBC
**$L_{foliage}\left(d,h\right)$**	15	0.942	0.940	5.80	2.40	0.189	417	206
**$L_{foliage}\left(d,h\right)$**	20	0.948	0.946	5.27	2.29	0.167	410	199

**Table 7:** Evaluation parameters for other greenhouses measurements. The greenhouses are locating in La Cañada, Retamar, El Alquián and Nijar. All these locations are at Almería province, Spain.

	Location	Year	R^2^	R^2^ Adj.	MSE	RMSE	MAPE	AIC	SBC
**$L_{foliage}\left(d,h\right)$**	Cañada	2019	0.921	0.920	8.745	2.957	0.408	445	234
**$L_{foliage}\left(d,h\right)$**	Retamar	2019	0.912	0.910	9.196	3.0325	0.261	449	237
**$L_{foliage}\left(d,h\right)$**	Alquián	2019	0.901	0.899	9.976	3.158	0.242	454	243
**$L_{foliage}\left(d,h\right)$**	Níjar	2019	0.895	0.892	11.486	3.389	0.602	464	253
**$L_{foliage}\left(d,h\right)$**	Cañada	2018	0.902	0.900	10.53	3.245	0.603	458	247
